# Development and validation of a nomogram prediction model based on albumin-to-alkaline phosphatase ratio for predicting the prognosis of gallbladder carcinoma

**DOI:** 10.3389/pore.2022.1610818

**Published:** 2023-01-04

**Authors:** Zizheng Fan, Bing Liu, Peizhong Shang

**Affiliations:** ^1^ Department of Graduate School, Hebei North University, Zhangjiakou, China; ^2^ Department of Hepatobiliary Surgery, The Hospital of 81st Group Army PLA, Zhangjiakou, China

**Keywords:** prognosis, nomogram, gallbladder carcinoma, independent risk factors, AAPR

## Abstract

Gallbladder carcinoma (GBC) is a rare biliary tract cancer with a high recurrence rate and a poor prognosis. Albumin-alkaline phosphatase ratio (AAPR) has been demonstrated to be a prognostic predictor for several cancers, but its predictive value for GBC patients remains unknown. The aim of this study was to investigate the predictive role of AAPR in GBC patients and to develop a novel nomogram prediction model for GBC patients. We retrospectively collected data from 80 patients who underwent surgery at the Hospital of 81st Group Army PLA as a training cohort. Data were collected from 70 patients with the same diagnosis who underwent surgery at the First Affiliated Hospital of Hebei North University as an external verification cohort. The optimal cut-off value of AAPR was determined using X-tile software. A nomogram for the overall survival (OS) based on multivariate Cox regression analysis was developed and validated using calibration curves, Harrell’s concordance index, the receiver operating characteristic curves, and decisive curve analyses. The optimal cut-off value of AAPR was .20. Univariate and multivariate Cox regression analyses demonstrated that BMI (*p* = .043), R0 resection (*p* = .001), TNM stage (*p* = .005), and AAPR (*p* = .017) were independent risk factors for GBC patients. In terms of consistency, discrimination, and net benefit, the nomogram incorporating these four independent risk factors performed admirably. AAPR is an independent predictor of GBC patients undergoing surgery, and a novel nomogram prediction model based on AAPR showed superior predictive ability.

## Introduction

Gallbladder carcinoma (GBC) is the most common malignant tumor of the biliary system, and its incidence ranks sixth among malignant tumors of the digestive system [1]. According to the latest GLOBOCAN 2020 database [2], the number of new cancer cases and cancer deaths in 2020 for GBC are 115,949 and 84,695, respectively. Due to the atypical clinical symptoms in the early stage, GBC is often misdiagnosed as biliary colic or chronic cholecystitis. Therefore, most GBC patients are in an advanced stage at the time of diagnosis. To date, radical resection remains the only effective treatment for patients with GBC [3]. The 5-year survival rate remains low due to high postoperative recurrence and metastasis rates [4]. Therefore, it is of great importance to establish a prognostic model for GBC patients to ensure timely treatment.

The concept of albumin-alkaline phosphatase ratio (AAPR) was originally proposed by Chan et al, who demonstrated that AAPR is an independent prognostic indicator of OS in hepatocellular carcinoma patients who underwent radical surgery [5]. In 2018, Tan et al. [6] proposed the predictive value of AAPR in patients with upper tract urothelial carcinoma. Subsequently, several studies demonstrated that AAPR is a clinical prognostic indicator for non-small cell lung cancer [7] and combined hepatocellular-cholangiocarcinoma [8]. Recently, a study was performed by Li et al. [9], who developed a nomogram model after radical cystectomy of bladder cancer, and considered that AAPR could be conducive to clinical decision-making and risk stratification in those patients. Nevertheless, AAPR, as a novel inflammation-related ratio index, has not been explored in GBC. Thus, it is valuable to study the potential application of AAPR in GBC.

This study aimed to investigate the prognostic value of AAPR in GBC patients and combine AAPR with other three independent risk factors to establish a nomogram prediction model for the prognosis of GBC patients.

## Patients and methods

### Patients

We retrospectively reviewed the medical records of GBC patients at the Hospital of the 81st Group Army PLA from January 2014 to May 2022 and the medical records of GBC patients at the First Affiliated Hospital of Hebei North University from March 2016 to March 2022. The inclusion criteria were as follows: 1) GBC confirmed by pathological examination; 2) Patients who underwent surgery; 3) No other malignant tumors; and 4) Complete medical records and follow-up data. Patients with incomplete medical records, missing follow-up data, or other malignant tumors were excluded from this study. This study was reviewed and approved by the ethics committee of the Hospital of 81st Group Army PLA (JTJYY-202207) and the First Affiliated Hospital of Hebei North University (W2021036).

### Data collection and definition

We collected clinical information of the two cohorts from the medical records including age, gender, body mass index (BMI), jaundice, gallbladder stone, diabetes mellitus, R0 resection, tumor location, tumor size, tumor number, tumor differentiation, TNM stage, Nevin stage, intraoperative blood loss, and survival time. Clinical staging was graded in accordance with the American Joint Committee on Cancer (AJCC) 8th edition of the GBC TNM staging system and the Nevin staging system. Albumin (ALB) and alkaline phosphatase (ALP) were obtained from preoperative blood biochemical tests. AAPR was the ratio obtained by dividing ALB by ALP.

### Statistical analysis

X-tile software (Rimm Laboratory, Yale School of Medicine, New Haven, CT, United States) was employed to determine the optimal cut-off value of AAPR [10]. The same method identified the optimal cut-off values for other associated factors. Differences in the unordered categorical variables and the ordinal categorical variables in the training cohort and verification cohort were determined using the Chi-square test and the Mann-Whitney U test, respectively. The relationship between AAPR and the clinical characteristics of patients was compared using the Chi-square test or Fisher’s exact test, as appropriate. The Kaplan-Meier method and the log-rank test were employed to calculate survival analyses. Univariate and multivariate Cox regression analyses were performed to analyze independent risk factors. A multicollinearity test was performed to assess the correlation between variables. Nomogram was constructed based on the results of multivariate Cox regression analysis, and the performance of the nomogram was evaluated using calibration curves, Harrell’s consistency index (C-index), the receiver operating characteristic (ROC) curves, and decisive curve analyses (DCA). In the present study, statistical analyses were carried out using the Statistical Package for the Social Sciences version 25.0 software (IBM Corp., Armonk, United States) and R software 4.2.0 (http://www.r-project.org/). *p*-value < .05 was considered statistically significant.

## Results

### Patients’ characteristics

The training cohort consisted of 60 (75.00%) females and 20 (25.00%) males, with a mean age of 65.95 ± 9.71 years. There were 52 (65.00%) patients with well or moderate cancer cell differentiation and 28 (35.00%) patients with poor cancer cell differentiation, respectively. According to the eighth edition of the AJCC TNM staging system, 3 (3.75%), 14 (17.50%), 3 (3.75%), 49 (61.25%), and 11 (13.50%) GBC patients were categorized as stage 0, I, II, III, and IV, respectively. The verification cohort included 53 (75.71%) females and 17 (24.29%) males, with a mean age of 64.11 ± 9.84 years. There were 35 (50.00%) patients with well or moderate cancer cell differentiation and 35 (50.00%) patients with poor cancer cell differentiation, respectively. According to the eighth edition of the AJCC TNM staging system, 7 (10.00%), 11 (15.71%), 3 (4.29%), 43 (61.43%), and 6 (8.57%) GBC patients were classified as stage 0, I, II, III, and IV, respectively.

In the training cohort, 43 (53.75%) patients died with a median OS of 20 months, The 1-, 3-, and 5-year OS rates were 64.40%, 36.60%, and 29.10%, respectively. In the verification cohort, 43 (61.43%) died with a median OS of 16 months. The 1- and 3-year OS rates were 57.90% and 30.10%, respectively. The clinicopathological characteristics of the patients in the training and verification cohorts were detailed in Table 1.

**TABLE 1:** Clinicopathological characteristics of 150 patients with gallbladder carcinoma in the training cohort and verification cohort.

Characteristics	Training cohort (%)	Verification cohort (%)	*P*-value
Number of patients	80	70	
Age, yr			.568
≤65	34 (42.50)	33 (47.14)	
>65	46 (57.50)	37 (52.86)	
Gender			.919
Male	20 (25.00)	17 (24.29)	
Female	60 (75.00)	53 (75.71)	
BMI, kg/m^2^			.563
≤25.3	55 (68.75)	45 (64.29)	
>25.3	25 (31.25)	25 (35.71)	
Jaundice			.598
No	58 (72.50)	48 (68.57)	
Yes	22 (27.50)	22 (31.43)	
Gallbladder stone			.349
No	31 (38.75)	22 (31.43)	
Yes	49 (61.25)	48 (68.57)	
Diabetes mellitus			.095
No	70 (87.50)	54 (77.14)	
Yes	10 (12.50)	16 (22.86)	
R0 resection			.453
No	31 (38.75)	23 (32.86)	
Yes	49 (61.25)	47 (67.14)	
Tumor differentiation			.063
Well or Moderate	52 (65.00)	35 (50.00)	
Poor	28 (35.00)	35 (50.00)	
TNM stage			.256
0	3 (3.75)	7 (10.00)	
I	14 (17.5)	11 (15.71)	
II	3 (3.75)	3 (4.29)	
III	49 (61.25)	43 (61.43)	
IV	11 (13.75)	6 (8.57)	
Nevin stage			
I	5 (6.25)	8 (11.43)	.261
II	13 (16.25)	17 (24.29)	
III	41 (51.25)	26 (37.14)	
IV	9 (11.25)	10 (14.28)	
V	12 (15.00)	9 (12.86)	
Tumor size, cm			.527
≤3	64 (80.00)	53 (75.71)	
>3	16 (20.00)	17 (24.29)	
Tumor location			.131
Body, bottom	72 (90.00)	57 (81.43)	
Neck, other	8 (10.00)	13 (18.57)	
Tumor number			.153
Single	67 (83.75)	52 (74.29)	
Multiple	13 (16.25)	18 (25.71)	
Intraoperative blood loss, ml			.965
≤50	46 (57.50)	40 (57.14)	
>50	34 (42.50)	30 (42.86)	
AAPR			.707
≤.20	24 (30.00)	23 (32.86)	
>.20	56 (70.00)	47 (67.14)	

Abbreviations: yr, year; BMI, body mass index; AAPR, albumin to alkaline phosphatase ratio.

### Relationship between AAPR and patients’ clinical characteristics in the training cohort

**FIGURE 1:**
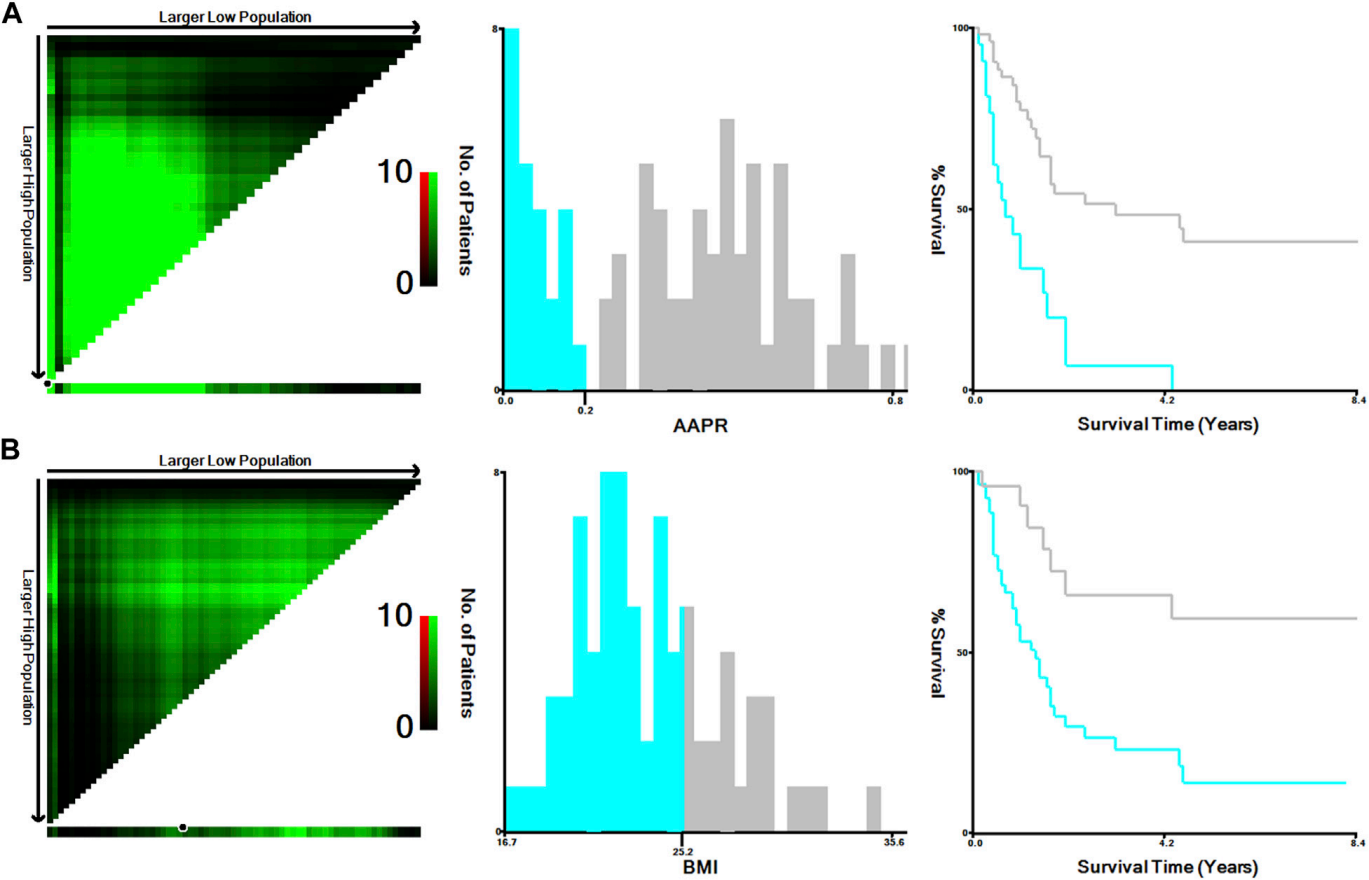
Calculation of optimal cut-off values of AAPR **(A)** and BMI **(B)** of the training cohort using X-tile software. Abbreviations: AAPR, albumin-to-alkaline phosphatase ratio; BMI, body mass index.

The optimal cutoff values for AAPR and BMI obtained with the X-tile software were 0.20 and 25.30, respectively (Figure 1). Patients were categorized into a low group (AAPR ≤ .20, *n* = 24) and a high group (AAPR > .20, *n* = 56) on the basis of the optimal cutoff value of AAPR. In the high AAPR group, the frequency of females was higher. Jaundice occurred more frequently in the low AAPR group compared to the high AAPR group, and the proportion of patients with R0 resection was greater in the high AAPR group than in the low AAPR group. The high AAPR group had a larger number of patients with tumor size less than 3 cm compared to the low AAPR group. Patients in the low AAPR group suffered more intraoperative blood loss than those in the high AAPR group. In the present study, AAPR seems to be not associated with BMI, TNM stage, and Nevin stage. The relationship between AAPR and other clinicopathological features is detailed in Table 2.

**TABLE 2:** Clinical characteristics of the patients according to albumin-to-alkaline phosphatase ratio in the training cohort.

Characteristics	AAPR≤.20, *n* (%)	AAPR>.20 *n* (%)	Χ^2^	*P*-value
Age, yr			.789	.374
≤65	12 (50)	22 (39.29)		
>65	12 (50)	34 (60.71)		
Gender			7.937	**.005**
Male	11 (45.83)	9 (16.07)		
Female	13 (54.17)	47 (83.93)		
BMI, kg/m^2^			1.732	.188
≤25.2	19 (79.17)	36 (64.29)		
>25.2	5 (20.83)	20 (35.71)		
Jaundice			53.608	**<.001**
No	4 (16.67)	54 (96.43)		
Yes	20 (83.33)	2 (3.57)		
Gallbladder stone			.123	.726
No	10 (41.67)	21 (37.50)		
Yes	14 (58.33)	35 (62.50)		
Diabetes mellitus			.136	.712
No	22 (91.67)	48 (85.71)		
Yes	2 (8.33)	8 (14.29)		
R0 resection			8.148	**.004**
No	15 (62.50)	16 (28.57)		
Yes	9 (37.50)	40 (71.43)		
Tumor differentiation			3.391	.066
Well or Moderate	12 (50)	40 (71.43)		
Poor	12 (50)	16 (28.57)		
TNM stage			1.270	.260
0–II	4 (16.67)	16 (28.57)		
III–IV	20 (83.33)	40 (71.43)		
Nevin stage			1.966	.161
I–II	3 (12.50)	15 (26.79)		
III–V	21 (87.50)	41 (73.21)		
Tumor size, cm			5.093	**.024**
≤3	15 (62.50)	49 (87.50)		
>3	9 (37.50)	7 (12.50)		
Tumor location			.800	.371
Neck	4 (16.67)	4 (7.14)		
Body, bottom	20 (83.33)	52 (92.86)		
Tumor number			.157	.692
Single	19 (79.17)	48 (85.71)		
Multiple	5 (20.83)	8 (14.29)		
Intraoperative blood loss, ml			11.263	**.001**
≤50	7 (29.17)	39 (69.64)		
>50	17 (70.83)	17 (30.36)		

Abbreviations: yr, year; BMI, body mass index; AAPR, albumin to alkaline phosphatase ratio. Indicate statistically significant.

**FIGURE 2:**
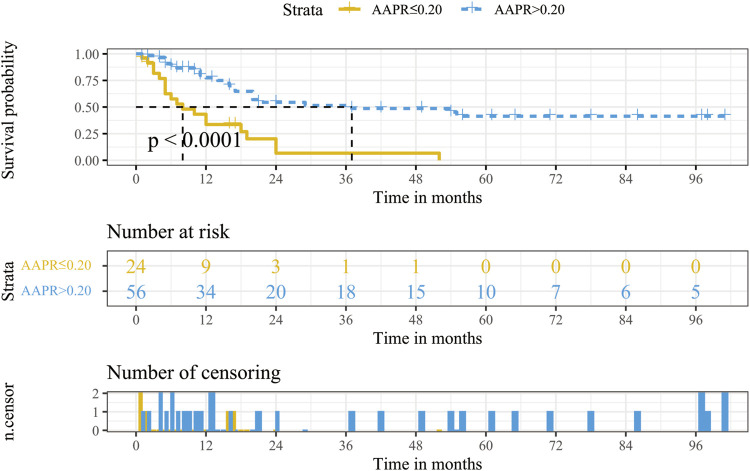
Kaplan-Meier survival analysis for OS stratified according to AAPR in the training cohort. Abbreviations: OS, overall survival; AAPR, albumin-to-alkaline phosphatase ratio.

The Kaplan-Meier curve of AAPR is presented in Figure 2. The median OS of the low AAPR group and the high AAPR group were 8 and 37 months, respectively (*p* < .0001).

### Independent prognostic factors in the training cohort

Univariate Cox analysis revealed that gender, jaundice, tumor differentiation, BMI, R0 resection, intraoperative blood loss, TNM stage, Nevin stage, tumor size, and AAPR were significantly associated with the prognosis of GBC patients (Table 3). The multicollinearity test revealed a strong relationship between the Nevin stage and the TNM stage (Tables 2, 3). As a result, the Nevin stage was excluded from the multivariate Cox model, and the TNM stage, which is more commonly used in clinical practice, was selected. The multivariate Cox analysis demonstrated that BMI [hazard ratio (HR) = .413, 95% confidence interval (CI): .175–.971; *p* = .043], R0 resection (HR = 3.096, 95%CI: 1.558–6.151; *p* = .001), TNM stage (HR = 4.921, 95%CI: 1.631–14.849; *p* = .005), and AAPR (HR = .212, 95%CI: .060–.757; *p* = .017) were independent risk factors for GBC patients (Table 3).

**TABLE 3:** Univariate and multivariate Cox proportional hazard analyses of factors associated with overall survival in the training cohort.

	Univariate test			Multivariate test		
	HR	95% CI	*P*-value	HR	95% CI	*P*-value
Age, yr						
≤65						
>65	1.040	.563–1.919	.901			
Gender						
Male						
Female	.522	.278–.978	**.043**	1.192	.580–2.446	.633
BMI, kg/m^2^						
≤25.2						
>25.2	.275	.121–.625	**.002**	.413	.175–.971	**.043**
Jaundice						
No						
Yes	2.853	1.521–5.350	**.001**	.512	.143–1.829	.303
Gallbladder stone						
No						
Yes	.853	.467–1.561	.607			
Diabetes mellitus						
No						
Yes	.727	.285–1.854	.505			
R0 resection						
Yes						
No	4.374	2.363–8.096	**<.001**	3.096	1.558–6.151	**.001**
Tumor differentiation						
Moderate-well						
Poor	2.079	1.136–3.805	**.018**	.905	.448–1.829	.781
TNM stage						
0–II						
III–IV	5.645	2.006–15.886	**.001**	4.921	1.631–14.849	**.005**
Nevin stage						
I–II						
III–V	5.477	1.944–15.430	**.001** ^a^			
Tumor size, cm						
≤3						
>3	2.369	1.204–4.663	**.013**	1.085	.487–2.415	.842
Tumor location						
Neck						
Body, bottom	.709	.252–1.994	.514			
Tumor number						
Single						
Multiple	1.158	.536–2.501	.708			
Intraoperative blood loss, ml						
≤50						
>50	3.011	1.621–5.595	**<.001**	1.645	.711–3.804	.245
AAPR						
≤.20						
>.20	.266	.142–.499	**<.001**	.212	.060–.757	**.017**

^a^ Nevin stage was excluded from the multivariate Cox regression due to the multicollinearity between the TNM, stage and the Nevin stage (VIF >5). Abbreviations: yr, year; BMI, body mass index; AAPR, albumin to alkaline phosphatase ratio. Indicate statistically significant.

### Establishment and verification of the nomogram

**FIGURE 3:**
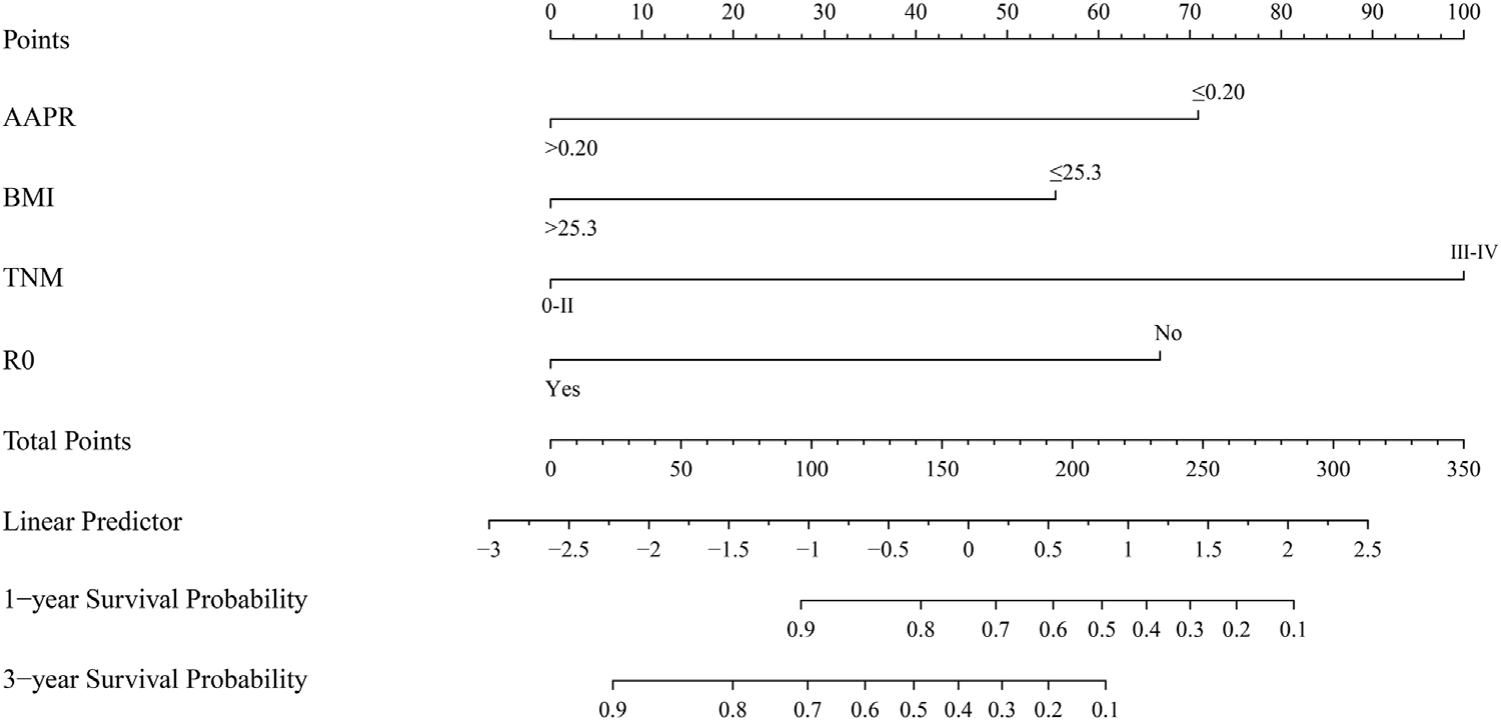
Nomogram based on AAPR, BMI, TNM stage, and R0 resection for predicting OS of GBC patients. Abbreviations: AAPR, albumin-to-alkaline phosphatase ratio; BMI, body mass index; OS, overall survival; GBC, gallbladder carcinoma.

**FIGURE 4:**
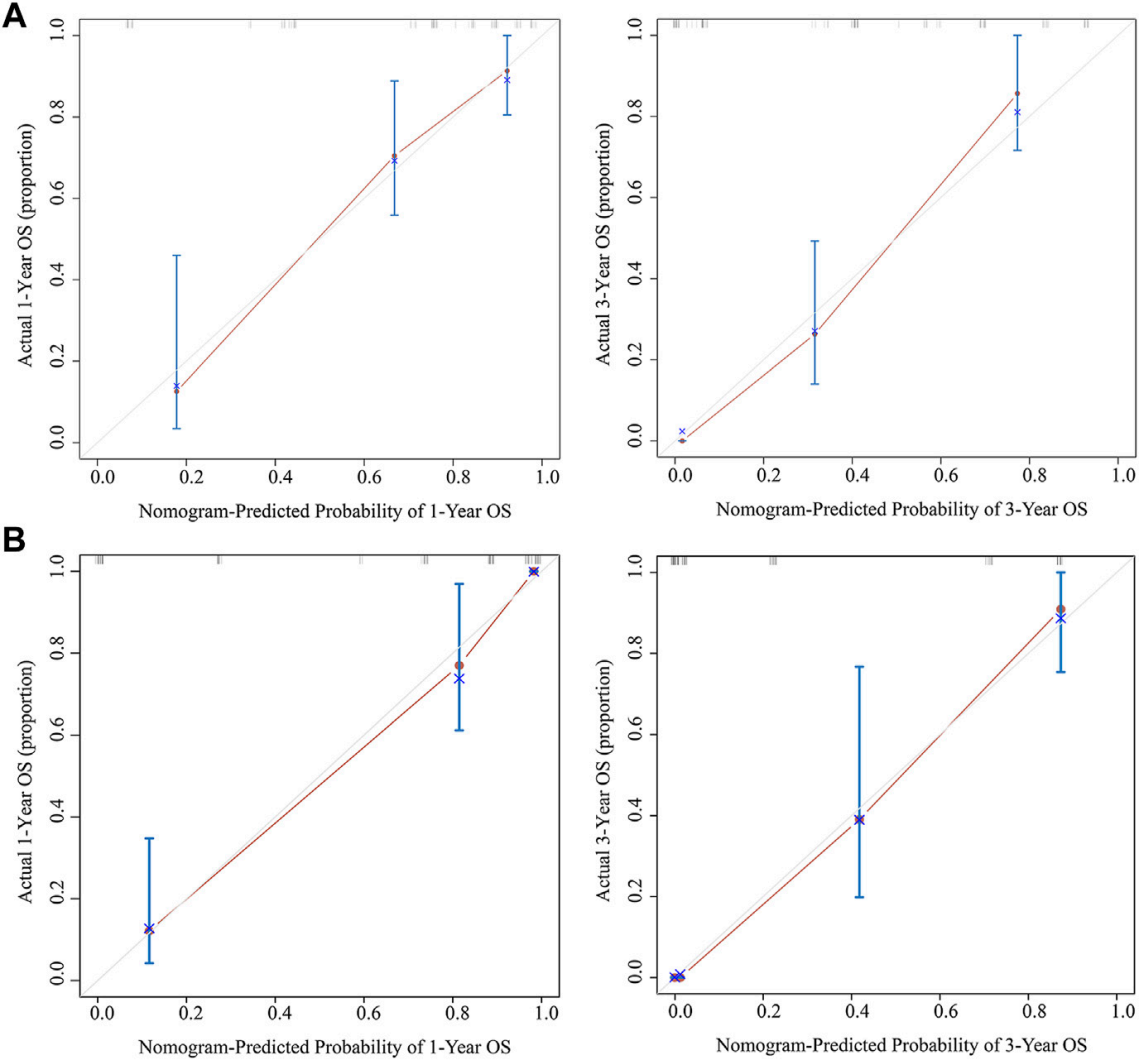
Calibration curves of the nomogram predicting 1- and 3-year OS for the training cohort **(A)** and verification cohort **(B)**. Abbreviation: OS, overall survival.

The results of multivariate Cox analysis showed that BMI, R0 resection, TNM stage, and AAPR were independent risk factors for predicting GBC patients after surgery (Table 3). Using the R software “RMS” package, a nomogram prediction model was developed by combining these four independent risk factors (Figure 3), and 1- and 3-year calibration curves of the training and verification cohorts were plotted, which demonstrated that results predicted by the nomogram prediction model were in good agreement with the actual observations in the calibration curves of the training and verification cohorts (Figure 4).

### Comparing different prediction models

**FIGURE 5:**
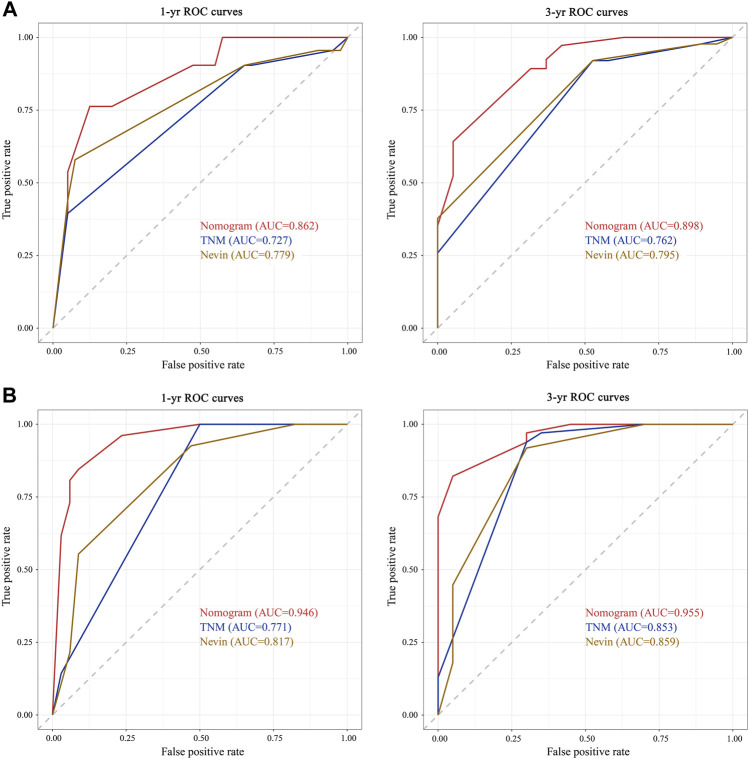
Comparisons of ROC curves of the nomogram prediction model, TNM staging system, and Nevin staging system for 1- and 3-year overall survival in the training cohort **(A)** and verification cohort **(B)**. Abbreviations: yr, year; AUC, area under the curve; ROC, receiver operating characteristic; OS, overall survival.

According to the ROC analysis in the training cohort, the area under the curve (AUC) at 1 year was .862, .727, and .779 for the nomogram prediction model, TNM staging system, and Nevin staging system, respectively, and the AUC at 3 years was .898, .762, and .795, respectively (Figure 5A). The C-index of the nomogram prediction model, TNM staging system, and Nevin staging system were .821, .716, and .745, respectively (Table 4). In the verification cohort, the nomogram prediction model’s 1- and 3-year AUCs were .946 and .955, respectively, which were significantly higher than those of the TNM and Nevin staging systems (Figure 5B). The AUCs and C-index in the training cohort and verification cohort were summarized in Table 4.

**TABLE 4:** Comparisons of the performance and discriminative ability between different prognosis prediction models in the training cohort and verification cohort.

	Training cohort			Verification cohort		
	Nomogram	TNM stage	Nevin stage	Nomogram	TNM stage	Nevin stage
1-yr AUC	.862	.727	.779	.946	.771	.817
3-yr AUC	.898	.762	.795	.955	.853	.859
C-index	.821	.716	.745	.888	.694	.690

Abbreviations: yr, year; AUC, area under the curve.

**FIGURE 6:**
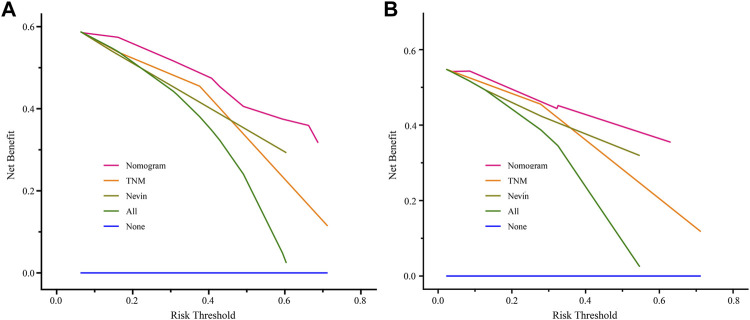
Comparisons of DCA of the nomogram prediction model, TNM staging system, and Nevin staging system for predicting 3-year survival probability in the training cohort **(A)** and verification cohort **(B)**. Abbreviation: DCA, decisive curve analysis.

In addition, to estimate the value of the clinical application of the nomogram prediction model, DCA was employed to compare the net benefit of the nomogram prediction model with the TNM and Nevin staging systems in the training cohort and the verification cohort (Figure 6). The results suggested that the nomogram prediction model generated more net benefit at 3 years after surgery in both the training and verification cohorts, across a wide range of threshold probabilities, which indicates that the nomogram prediction model may have great potential and application in clinical practice.

### The Establishment of the risk stratification model

**FIGURE 7:**
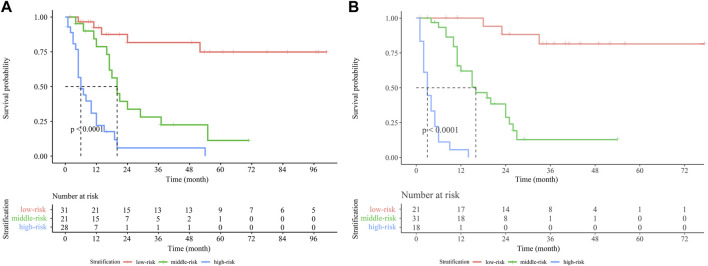
Kaplan-Meier survival curves for GBC patients stratified by the nomogram score in the training cohort **(A)** and verification cohort **(B)**. Abbreviation: GBC, gallbladder carcinoma.

In the present study, a novel risk stratification model was developed based on the total nomogram score of each patient in the training cohort and verification cohort. Patients in both cohorts were divided into the low-risk, middle-risk, and high-risk groups, respectively. In the training cohort, the median OS of the middle-risk and high-risk groups was 20 and 6 months, respectively (Figure 7A). In the verification cohort, the median OS was 16 and 3 months for the middle-risk and high-risk groups, respectively (Figure 7B). Patients in the high-risk group had a lower OS rate than those within the low-risk or middle-risk group, indicating that the risk stratification model based on the nomogram was also predictive.

## Discussion

GBC is a rare malignancy of the gastrointestinal tract, accounting for 1.2% of all malignancies and 50% of biliary tract malignancies worldwide [11]. Because of the late presentation of clinical manifestations and poor prognosis, it is often at an advanced stage at the time of diagnosis, and the 5-year survival rate for GBC is 5%–10% [12]. Therefore, accurately predicting the prognosis of GBC helps clinical decision-making for individualized postoperative treatment and then using the targeted treatment for different patient groups after surgery, which in turn improves the prognosis of patients. In this study, we aimed to evaluate the predictive value of AAPR and to develop a prognostic prediction model as a nomogram for GBC patients. This nomogram can be used as a reference for clinical decision-making and patient risk stratification, which has promising prospects for clinical use.

Serum ALB is a protein synthesized in the liver, consisting of 585 amino acid residues, which plays an important role in maintaining oncotic pressure, transporting nutrients, and other microenvironmental systems. Recent studies have revealed the ability of ALB in stabilizing cell proliferation, DNA replication, and exerting antioxidant reactions for anti-carcinogenesis [13–15]. Meanwhile, ALB is an important indicator to assess the nutritional status of the organism and can also reflect the systemic inflammatory response of the patient. Malnutrition can affect the body’s defense mechanisms, which in turn increases tumorigenesis [16]. Previous studies have illustrated that ALB has anti-inflammatory and immunomodulatory attributes and that inflammation-induced reductions in ALB synthesis may have an impact on immune defense [17, 18]. The body’s inflammatory response is closely related to carcinogenesis, tumor progression, and metastasis, and inflammatory factors cause damage to the body and promote the malignant progression of tumors. A growing number of studies have shown that serum ALB levels are associated with poor prognosis in a variety of malignancies [19–21]. Wheler et al. [22] discovered that low serum ALB levels in patients with metastatic breast cancer were significantly associated with shorter survival. And another study found that endometrial cancer patients with low serum ALB levels had a significantly shorter survival time [23]. Furthermore, a recently published study found that low serum ALB levels predicted a worse prognosis for patients with metastatic gastric cancer [24]. ALP, mainly concentrating in the liver, kidney, and bones, is a hydrolytic enzyme widely distributed in the body. A previous study has shown that ALP plays an important role in enhancing cancer cell proliferation, vascular invasion, and distant metastasis [25]. In addition, ALP has also been reported as an independent prognostic factor in patients with hepatocellular carcinoma, skeletal metastatic nasopharyngeal carcinoma, hypertrophic cardiomyopathy, and periampullary carcinoma [26–29]. AAPR is the ratio of ALB to ALP. Several studies have demonstrated that low AAPR is strongly associated with poor prognosis in malignancies [6, 30–33].

Currently, the prognostic indicators of GBC commonly used in clinical practice, such as the TNM staging system, Nevin staging system, and the histological grade, are mainly obtained from postoperative examination of tumor tissues. Although widely employed in clinical practice, the AJCC TNM staging system has inherent drawbacks. Because it only takes the conditions of the primary tumor, lymph nodes, and metastasis into consideration and lacks a personal examination reference for individual patients. According to a recent study, a nomogram prediction model based on the albumin-γ-glutamyl transferase ratio outperformed the TNM staging system [34]. Unfortunately, the Nevin staging system was not included in the study. In this study, we developed a novel nomogram prediction model based on AAPR and compared it with TNM staging system and Nevin staging system. The results of the ROC curve, C-index, and DCA revealed that the nomogram prediction model based on AAPR had significantly better predictive performance than the TNM staging system and the Nevin staging system, as well as greater clinical application value.

In the present study, we discovered that AAPR, a novel inflammation-related ratio index defined by preoperative levels of ALB and ALP, is an independent risk factor for GBC patients, with AAPR levels less than .20 having a lower OS. Previous studies have shown that AAPR is an excellent predictor of the prognosis of several types of cancer. However, its value in GBC has not been reported yet. To the best of our knowledge, this is the first study analyzing the predictive value of AAPR in GBC patients who received surgery. This study found that AAPR was associated with several clinicopathological characteristics, such as gender, jaundice, R0 resection, tumor size, and intraoperative blood loss. A high level of AAPR indicates a better prognosis and a longer OS. GBC patients with AAPR high-level had significantly higher 1- and 3-year OS rates than GBC patients with AAPR low-level. Meanwhile, we integrated AAPR with the other three independent prognostic factors, including BMI, R0 resection, and TNM stage, to develop a predictive nomogram for 1-, and 3-year survival probability. The nomogram achieved considerable prognostic performance in terms of consistency, discrimination, and net benefit, and AAPR was demonstrated to have superior predictive ability for GBC patients. The current nomogram also showed better discrimination and more net benefit compared with TNM and Nevin staging systems for GBC patients. In addition, we developed a risk stratification model according to the nomogram to assist clinicians in identifying GBC patients at high risk and provide early warning for a better individualized and precise treatment.

In this study, we demonstrated for the first time the prognostic value of AAPR in GBC patients and validated using external data. However, this study has some limitations. First, it was a retrospective study. Second, due to the fact that GBC is a relatively rare malignancy, the sample size enrolled was relatively limited. Finally, AAPR as a potentially valuable prognostic indicator needs to be further confirmed in future prospective studies for its prognostic value in GBC.

## Conclusion

In summary, our study revealed that the AAPR was an independent predictive factor for the prognosis of GBC patients. According to this finding, we developed a nomogram based on AAPR for a prognostic prediction model and further clinical decision-making in GBC patients.

## Data Availability

The original contributions presented in the study are included in the article/Supplementary Material, further inquiries can be directed to the corresponding author.
