# Supplementary material for: Development and validation of a nomogram prediction model based on albumin-to-alkaline phosphatase ratio for predicting the prognosis of gallbladder carcinoma
Source: Pathol Oncol Res. 2023 Jan 4;28:1610818. doi: 10.3389/pore.2022.1610818 (PMC9845243; doi:10.3389/pore.2022.1610818)
Supplement: Supplementary file 1 [file Table1.docx]

**Supplementary Table**

**Table S1. Multicollinearity test based on univariate Cox analysis results in the training cohort**

|  | **Tolerance** | **VIF** |
| --- | --- | --- |
| Gender | 0.790 | 1.267 |
| BMI, kg/m^2^ | 0.878 | 1.139 |
| Jaundice | 0.279 | 3.589 |
| R0 resection | 0.761 | 1.314 |
| Tumor differentiation | 0.818 | 1.223 |
| TNM stage | 0.109 | 9.142 |
| Nevin stage | 0.111 | 8.986 |
| Tumor size, cm | 0.829 | 1.207 |
| Intraoperative blood loss, ml | 0.727 | 1.376 |
| AAPR | 0.275 | 3.631 |

Abbreviations: VIF, variance inflation factor; BMI, body mass index; AAPR, albumin to alkaline phosphatase ratio.
